# Unusual Armadillo Fold in the Human General Vesicular Transport Factor p115

**DOI:** 10.1371/journal.pone.0004656

**Published:** 2009-02-27

**Authors:** Harald Striegl, Yvette Roske, Daniel Kümmel, Udo Heinemann

**Affiliations:** 1 Max-Delbrück-Centrum für Molekulare Medizin, Berlin, Germany; 2 Institut für Chemie und Biochemie, Freie Universität Berlin, Berlin, Germany; Griffith University, Australia

## Abstract

The golgin family gives identity and structure to the Golgi apparatus and is part of a complex protein network at the Golgi membrane. The golgin p115 is targeted by the GTPase Rab1a, contains a large globular head region and a long region of coiled-coil which forms an extended rod-like structure. p115 serves as vesicle tethering factor and plays an important role at different steps of vesicular transport. Here we present the 2.2 Å-resolution X-ray structure of the globular head region of p115. The structure exhibits an armadillo fold that is decorated by elongated loops and carries a C-terminal non-canonical repeat. This terminal repeat folds into the armadillo superhelical groove and allows homodimeric association with important implications for p115 mediated multiple protein interactions and tethering.

## Introduction

Membrane trafficking in eukaryotic cells is an example for the modular organization of cellular activity. The formation and delivery of transport intermediates to specific cellular locations are complex processes that can be divided into several stages [1]. In this modular organization the first interaction of a vesicle and its target membrane is termed tethering. It depends on a heterogeneous group of proteins called ‘tethers’ [2]. They can be divided into multi-subunit tethering complexes and proteins containing an extended coiled-coil region.

The golgin p115, which forms stable homodimers, is recruited to membranes in a nucleotide-dependent manner by the guanosine triphosphatase (GTPase) Rab1a [2], [3] and belongs to the family of tethers containing an extended coiled-coil region. p115 is among the best characterized representatives of long coiled-coil tethers. The architecture of p115 comprises a long central coiled-coil region, a large globular N-terminal domain and a C-terminal acidic region. The central region mediates homodimerization and contains the Rab1a binding site. Interaction of Rab1a and p115 is thought to tether coat-protein complex II (COP II) vesicles to each other, thus promoting homotypic vesicle fusion [3]. The C-terminal region of p115 binds to GM130 and giantin, two further coiled-coil tethers localized at the Golgi membrane [4].

p115 binds to a specific set of soluble N-ethylmaleimide-sensitive-factor attachment protein receptors (SNAREs), aiding formation of a *cis*-SNARE complex that promotes the antero-grade ER-to-Golgi transport by targeting COP II vesicles to the Golgi apparatus [3]. In addition to its major role in exocytotic transport (5), p115 functions in retrograde Golgi-to-ER trans-port, intra-Golgi transport and Golgi biogenesis [6] of the Golgi apparatus, due to essential interactions with the coat-protein complex I (COP I) subunit β-COP [7] and the conserved-oligomeric-Golgi complex (COG) subunit COG2 [8].

**Figure 1:**
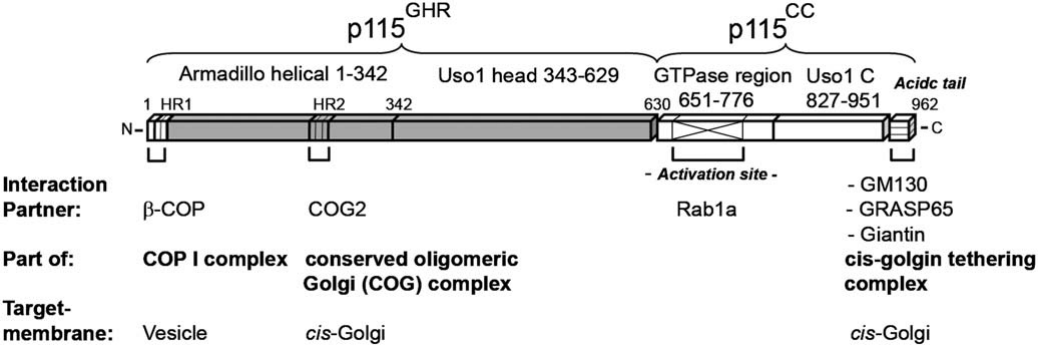
Schematic overview of full-length p115. The construct comprising p115^GHR^ used for crystallization is shown in gray.

To understand how these different activities are combined in one p115 molecule, we embarked on its structure analysis. We used a construct comprising the globular head region of p115 (p115^GHR^, residues Asp54 to Tyr629) for crystallization (Fig. 1). The fragment lacks 53 N-terminal residues that are predicted to be disordered [9] and the C-terminal coiled-coil domain (p115^CC^).

## Results and Discussion

### Structure of the p115 globular head region

**Figure 2:**
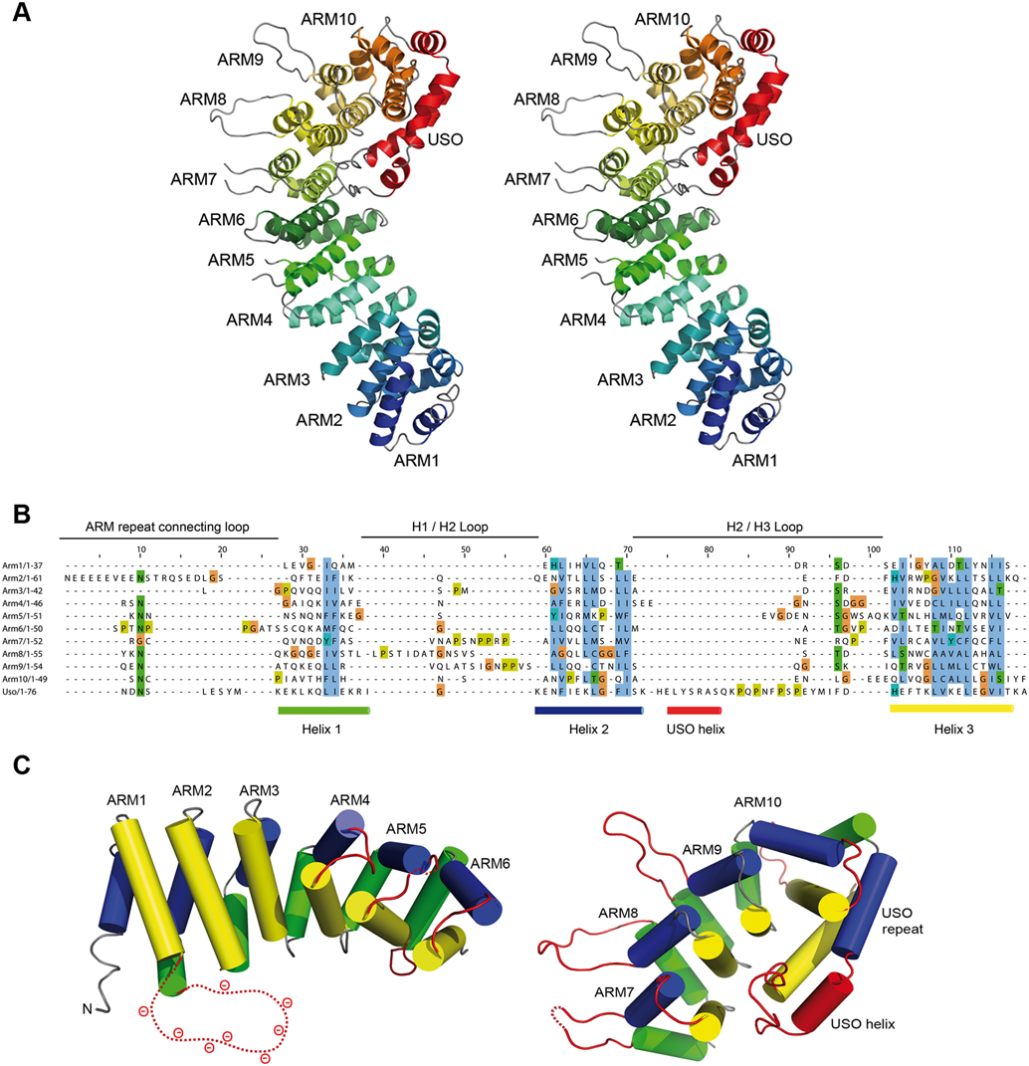
Crystal structure of p115^GHR^. (A) Stereo view of the overall structure. The protein is composed of 11 armadillo repeats. (B) Structure-based sequence alignment of the p115 armadillo repeats and the loop regions. Consensus residues that define the conserved hydrophobic residues of each armadillo repeat are highlighted in blue; amino acids that define conserved polar, neutral residues are highlighted in green. Glycine and proline residues are highlighted in brown and olive, respectively. The repeat numbers are shown on the left. The sequences that form helices H1, H2, and H3 are indicated as green, blue and yellow cylinders. The corresponding ARM loops are marked on top, the USO helix is indicated as red cylinder. (C) N-terminal armadillo-like helical domain (left) and C-terminal Uso1 head domain (right) of p115^GHR^, elongated loops with at least 5 residues are colored in red (H1 green, H2 blue, H3 yellow).

p115^GHR^ consists of a multi-helical β-catenin-like armadillo fold arranged in a regular right-handed superhelix (Fig. 2A). We observe 10 classical armadillo repeats (ARM1-ARM10) [10]–[12] and one non-canonical repeat which we termed USO repeat, after the yeast homolog of p115, Uso1p. Each armadillo repeat is composed of three α-helices (H1–H3) and has a distinct hydrophobic core. ARM1 and ARM2 are connected by a highly acidic and flexible loop (residues 92–110), which is not visible in the electron density. Structure-based sequence alignment reveals a number of highly conserved amino acids (Fig. 2B).

The N-terminal region of p115^GHR^ (Fig. 2C left) is remarkably similar to other armadillo-fold proteins [11], [13] of different subfamilies (β-catenin, p120/catenin δ-1, and karyopherin-α/importin-α), although these proteins show low sequence conservation. The C-terminal region (ARM7-USO) of p115^GHR^ differs from other members of armadillo-protein subfamilies (Fig. 2C right). The armadillo repeats exhibit long loops (5 to 13 residues) in ARM5-ARM9. This structural motif of elongated loops culminates in the formation of a short helix inserted in the H2-H3 loop (37 residues) of the terminal USO repeat which we named the USO helix. This USO repeat does not follow the rule of classical armadillo repeat proteins that form a right-handed superhelix. It folds back into the superhelical groove, leading to a globular C-terminus of p115^GHR^ and covering helices H3 of ARM8-ARM9, while the USO helix points into the center of the groove.

These unique characteristics of the C-terminus allow to structurally separate the protein in an armadillo helical domain (ARM1-ARM6, residues 54–342) and an Uso1 head domain (ARM7-USO, residues 343–629), which clearly distinguishes the head region of p115 from any other armadillo-fold protein. The Uso1 head domain identifies a group of proteins which are described as general vesicular transport factors, transcytosis associated proteins (TAP) or vesicle docking proteins [14].

### Interaction of p115^GHR^ and the COG complex subunit COG2

**Figure 3:**
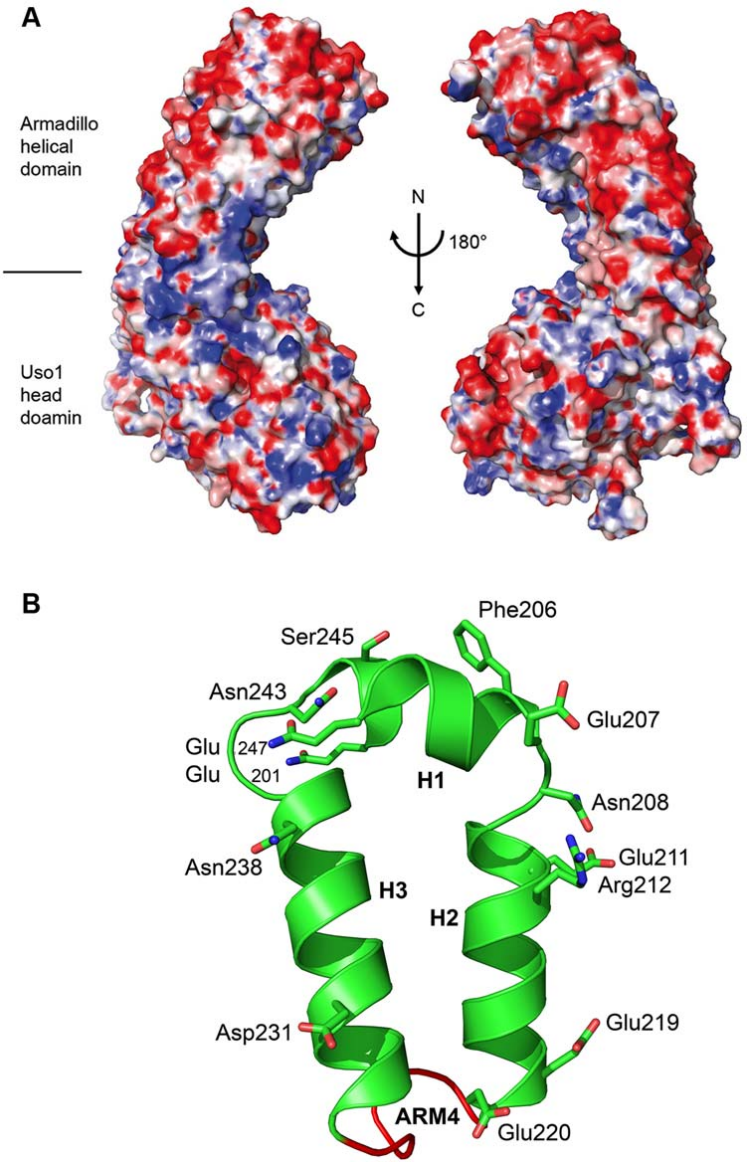
Interaction of p115^GHR^ and the COG complex subunit COG2. (A) Electrostatic surfaces of p115^GHR^. The amino terminus of the molecule is at the top of the figure. Blue indicates positive charge and red negative charge at the level of 10 kT/e. (B) Conserved residues of p115 and the yeast homolog Uso1p form a highly charged surface of exposed helices which define the COG2 binding site.

Uso1p and p115 share a similar domain structure, a large globular head region with a long coiled-coil domain and an acidic patch on the C-terminus of the protein with an overall sequence identity of 25%. Two highly conserved homologous regions HR1 (residues 21–54) and HR2 (residues 200–247) were shown to bind to the appendix domain of the COP I subunit β-COP and the COG complex subunit COG2, respectively (see Fig. 1). HR1 is predicted unordered and missing in our structure. The HR2 is mapped to ARM4 and ARM5 of the N-terminal armadillo like helical domain. The armadillo fold is found in more than 240 proteins that mostly serve as scaffolds for the assembly of multiprotein complexes. They often mediate complex formation by polar interactions. Interestingly, the armadillo helical domain shows large negatively charged patches (Fig. 3a), and additionally we observe a conserved, highly charged surface patch of ARM4 in HR2 [15], [16] which indicates that COG2 binding arises mainly from polar interactions [Fig. 3b].

### Dimeric arrangement of the p115 globular head region

**Figure 4:**
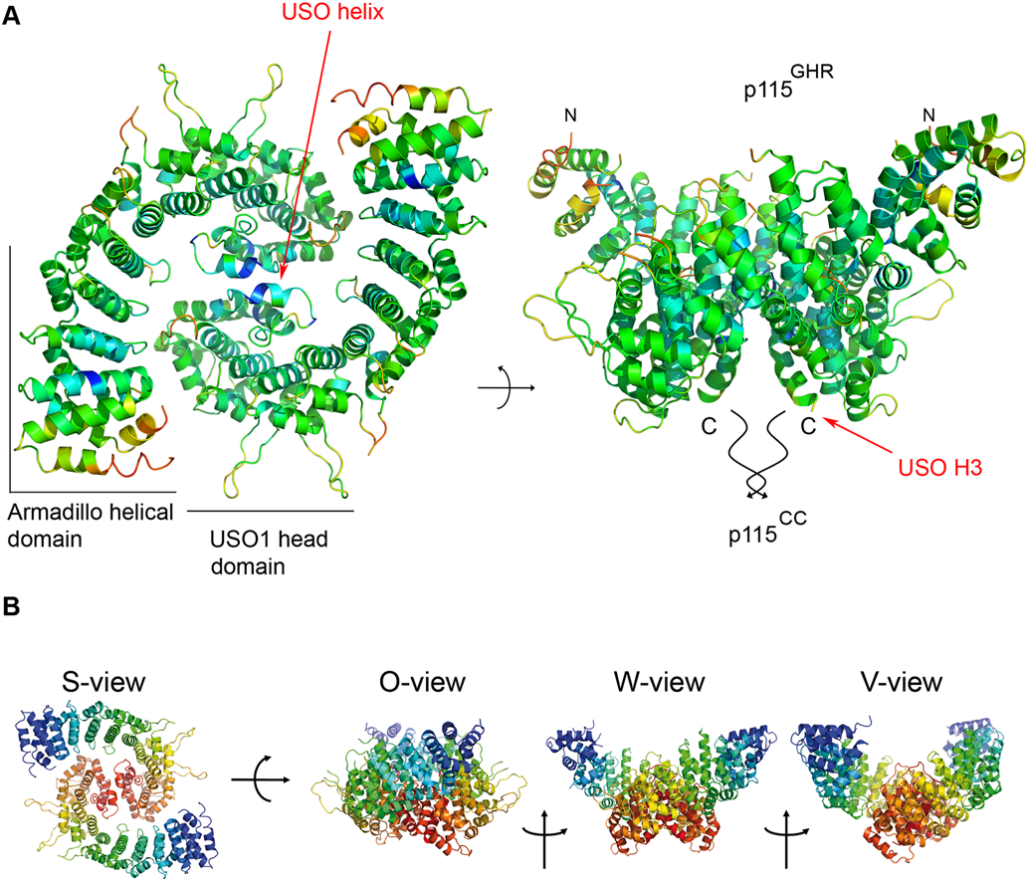
Dimeric arrangement of the p115 globular head region. (A) *B*-factor representation (“S-“ and “W-view”) of p115^GHR^ molecules aligned by crystal symmetry. The intermolecular contact area (blue) is among the most rigid parts of the structure. (B) Depending on the orientation, the crystallographic dimer of p115^GHR^ has a single-head (“O-view”) or double-lobed globular appearance (“W-” and ”V-view”).

**Figure 5:**
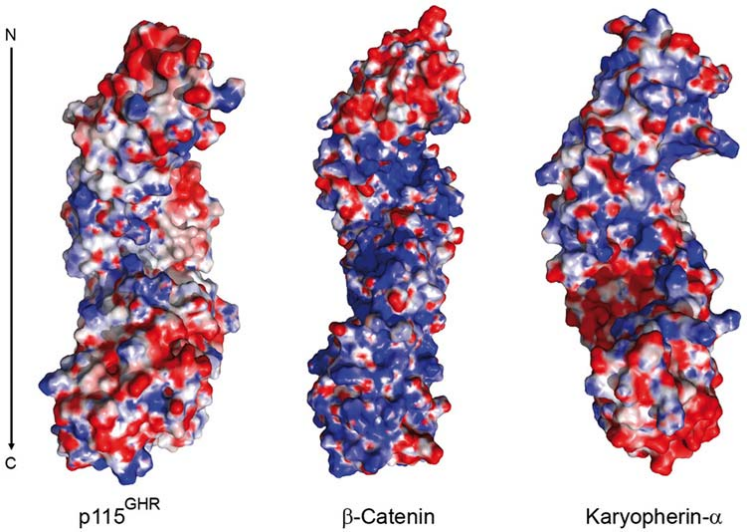
Electrostatic surfaces comparison of the superhelical grooves of the p115^GHR^, β-catenin and karyopherin-α. Blue indicates positive charge and red negative charge at the level of 10 kT/e. The amino terminus of the molecule is at the top of the figure.

p115, like other golgins, is a stable homodimer with an N-terminal globular head domain and a C-terminal coiled-coil domain of 45 nm length as determined by rotary-shadowing electron microscopy [17]. We have observed p115^GHR^ to be monomeric in solution by gel-filtration experiments (not shown). In the crystal structure, a dimeric arrangement between p115^GHR^ molecules results from their packing along a dyad axis (Fig. 4A). Depending on the orientation, the crystallographic dimer has a single-head or double-lobed globular appearance (Fig. 4B). The extended loops point towards the exposed surface, and the large superhelical groove of one molecule is covered by the groove of the second molecule in the dimeric arrangement. Interestingly, the p115^GHR^ groove is less charged compared to β-catenin and karyopherin-α (Fig. 5) which there serves as a binding site for interaction partners in these proteins. In the dimeric p115^GHR^ assembly as observed in the crystal the monomers are twisted around each other, keeping the USO helices, which form the interface of the head dimer, in the center. The USO helix and the USO repeat helix H2 are part of the dimer interface which covers only 635 Å^2^ (∼2.6%) of the total 24,000 Å^2^ of solvent-accessible surface (SAS). This contact area is relatively small, indicating that in solution the globular head domains might be connected flexibly, if at all.

### A Model for p115 full length protein

**Figure 6:**
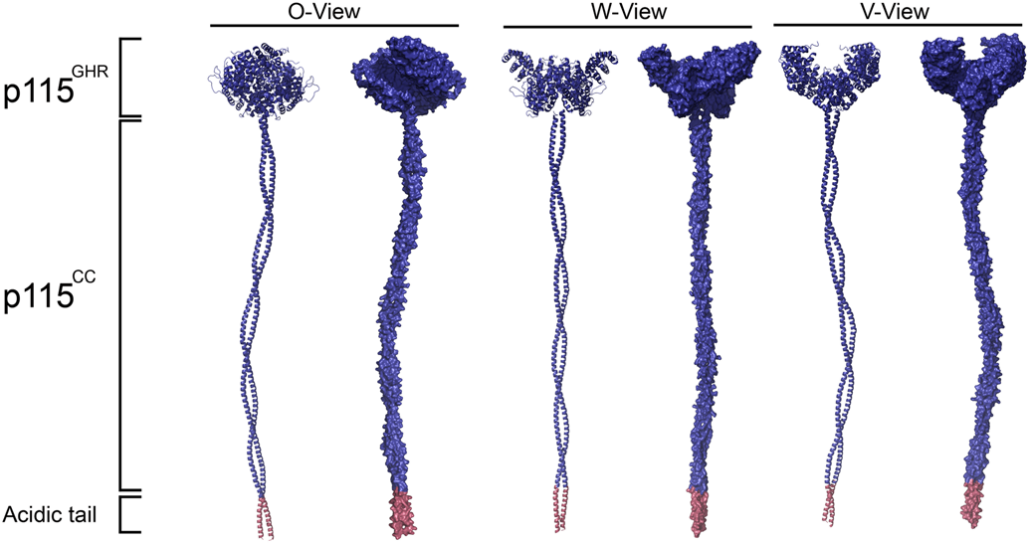
Model of the overall fold of the full-length p115. Surface and cartoon representations of a model of the full-length general vesicular transport factor p115 generated by manually fitting a coiled-coil of appropriate length to the C-termini of p115^GHR^ in the crystallographic dimer. The different views of the p115 model closely resemble published electron micrographs of p115 and Uso1p.

Although the observed crystallographic dimer might not exactly reflect the protein structure in the cell, we suggest a model of the overall fold of the full-length p115 (Fig. 6). We note the distinct shape similarity between the dimer arrangement of p115^GHR^ and EM images of intact dimeric p115 [17] and Uso1p [18]. In agreement with this observation, the C-termini of both p115^GHR^ monomers are aligned in parallel in the crystal structure which would allow continuation into the coiled-coil of p115.

The different members of armadillo subfamilies like β-catenin, karyopherin-α and p115^GHR^ define a conserved architecture and provide a scaffold for the assembly of protein complexes with different functions. Interestingly, the C-terminal region of p115^GHR^, in comparison to full-length β-catenin [19] shows how the architecture of an armadillo domain is altered to serve in, what we propose, a hinge-linkage between the subunits of the dimeric p115 head domain. Further high-resolution structures of p115^GHR/CC^ and binding partner complexes combined with characterization of structure based mutants in cell-based assays will be required to understand how p115 carries out its tethering function.

## Materials and Methods

### Protein expression and purification

A fragment of the human *p115* gene, encoding amino-acid residues 54–628 (p115^GHR^), was cloned into the bacterial expression vector pGEX-4T1 (GST Gene Fusion System, GE Healthcare) and expressed in Superior Broth (SB) medium with 1 mM isopropyl-1-thio-β-D-galactopyranoside. p115^GHR^ was purified with GST-affinity chromatography subjected to size-exclusion chromatography after tag cleavage by thrombin and concentrated to 20.0 mg ml^−1^. Selenomethionine-labeled p115 was produced by using metabolic inhibition of the methionine pathway according to the protocol of Van Duyne *et al.*, [20].

### Crystallization and data collection

p115^GHR^ crystals were grown at 4°C by the sitting-drop method using a semi-automated dispensing system [21]. Crystals for X-ray measurements were obtained in 25% PEG 550 MME, 0.1 M HEPES pH 7.5. The best crystals were flash-cooled at 100 K in mother liquor containing 20% sucrose. Data from a native crystal to 2.2 Å and a crystal from selenium-labeled p115^GHR^ to 2.8 Å resolution were collected at 100 K at the Protein Structure Factory beamline 14.2 of the Freie Universität Berlin [21] at BESSY (Berlin, Germany). The same space group, C2, was obtained for the native and selenomethionyl proteins, with one molecule per asymmetric unit. Data were reduced and scaled using HKL2000 [22]. Data collection statistics are listed in Table 1.

**Table 1:** Data collection and refinement statistics.

	Native p115^GHR^	Selenomethionyl p115^GHR^
**Data collection**		
Resolution (Å)	19.85-2.2	83.62-2.8
Wavelength (Å)	0.91841	0.97965
Images	1–160	1–130
Detector	MarCCD 165 mm	MarCCD 165 mm
Space group	C2	C2
Observed reflections	145,857	59,377
Independent reflections	43,877	21,841
<*I*/σ(*I*)>	10.2	17.3
Redundancy	3.3	2.7
Completeness: ov./l.s. (%)	90.5/82.5	98.5/98.2
Cell *a*, *b*, *c* (Å)	175.55, 68.89, 85.75	179.56, 63.09, 85.68
α, β, γ (°)	90, 108.74, 90	90, 111.15, 90
*R*_sym_ (%): ov./l.s	6.7/43.0	6.4/22.3
**Refinement**		
*R*_work_/*R*_free_ (%)	21.94/26.94	
Number of non-hydrogen atoms	4439	
Number of water molecules	123	
rms deviation from ideal geometry:		
Bond lengths (Å)	0.012	
Bond angles (°)	1.45	
Torsion angles (°)	5.67	
Number of residues	553	
Overall mean *B* value (Å^2^)	43.39	
Ramachandran statistics:		
Residues in favored regions (%)	95.4 (526/549)	
Residues in allowed regions (%)	100 (549/549)	
Residues in disallowed regions (%)	-	

### Structure determination and refinement

For structure determination of p115^GHR^, selenium-peak wavelength data to 2.8 Å resolution were used for single-wavelength anomalous diffraction phasing (SAD) to determine the positions of 15 selenium sites. Initial phases were calculated and improved using PHENIX [23]. The initial model was automatically built with ARP/wARP [24] and manually improved using the program COOT [25]. The model was placed into the unit cell of the higher-resolution native protein and subsequently refined using REFMAC5 [26]. During several rounds of iterative model building and refinement (including TLS), the model was extended to 553 residues per asymmetric unit, and three polyethylene glycol and 123 water molecules were placed into the electron density. The p115^GHR^ structure has a final *R*_work_ = 21.9% and *R_free_* = 26.9%, and the quality of the model was excellent as assessed with the program Molprobity [27]. The coordinates and diffraction amplitudes were deposited in the Protein Data Bank with accession code 2w3c. Refinement statistics are summarized in Table 1.

### Figure production

All pictures were prepared using PyMOL [28] and the APBS tool [29]. The sequence alignment was prepared with ClustalW [30].

## References

[1] Hofmann KP, Spahn CM, Heinrich R, Heinemann U (2006). Building functional modules from molecular interactions.. Trends Biochem Sci.

[2] Allan BB, Moyer BD, Balch WE (2000). Rab1 recruitment of p115 into a cis-SNARE complex: programming budding COPII vesicles for fusion.. Science.

[3] Beard M, Satoh A, Shorter J, Warren G (2005). A cryptic Rab1-binding site in the p115 tethering protein.. J Biol Chem.

[4] Shorter J, Warren G (1999). A role for the vesicle tethering protein, p115, in the post-mitotic stacking of reassembling Golgi cisternae in a cell-free system.. J Cell Biol.

[5] Satoh A, Warren G (2008). In situ cleavage of the acidic domain from the p115 tether inhibits exocytic transport.. Traffic.

[6] Puthenveedu MA, Linstedt AD (2004). Gene replacement reveals that p115/SNARE interactions are essential for Golgi biogenesis.. Proc Natl Acad Sci USA.

[7] Guo Y, Punj V, Sengupta D, Linstedt AD (2008). Coat-tether interaction in Golgi organization.. Mol Biol Cell.

[8] Sohda M, Misumi Y, Yoshimura S, Nakamura N, Fusano T (2007). The interaction of two tethering factors, p115 and COG complex, is required for Golgi integrity.. Traffic.

[9] Li X, Romero P, Rani M, Dunker AK, Obradovic Z (1999). Predicting protein disorder for N-, C-, and internal regions.. Genome Informatics.

[10] Huber AH, Nelson WJ, Weis WI (1997). Three-dimensional structure of the armadillo repeat region of β-catenin.. Cell.

[11] Riggleman B, Wieschaus E, Schedl P (1989). Molecular analysis of the armadillo locus: uniformly distributed transcripts and a protein with novel internal repeats are associated with a Drosophila segment polarity gene.. Genes Dev.

[12] Peifer M, Berg S, Reynolds A (1994). A repeating amino acid motif shared by proteins with diverse cellular roles.. Cell.

[13] Hatzfeld M, Nachtsheim C (1996). Cloning and characterization of a new armadillo family member, p0071, associated with the junctional plaque: evidence for a subfamily of closely related proteins.. J Cell Sci.

[14] Apweiler R, Attwood TK, Bairoch A, Bateman A, Birney E (2000). The InterPro database, an integrated documentation resource for protein families, domains and functional sites.. Nucleic Acids Res.

[15] Fernandez-Recio J, Totrov M, Skorodumov C, Abagyan R (2005). Optimal docking area: a new method for predicting protein-protein interaction sites.. Proteins.

[16] Glaser F, Pupko T, Paz I, Bell RE, Bechor-Shental D, Martz E, Ben-Tal N (2003). ConSurf: identification of functional regions in proteins by surface-mapping of phylogenetic information.. Bioinformatics.

[17] Sapperstein SK, Walter DM, Grosvenor AR, Heuser JE, Waters MG (1995). p115 is a general vesicular transport factor related to the yeast endoplasmic reticulum to Golgi transport factor Uso1p.. Proc Natl Acad Sci USA.

[18] Yamakawa H, Seog DH, Yoda K, Yamasaki M, Wakabayashi T (1996). Uso1 protein is a dimer with two globular heads and a long coiled-coil tail.. J Struct Biol.

[19] Xing Y, Takemaru K, Liu J, Berndt JD, Zheng JJ, Moon RT, Xu W (2008). Crystal structure of a full-length beta-catenin.. Structure.

[20] Van Duyne GD, Standaert RF, Karplus PA, Schreiber SL, Clardy J (1993). Atomic structures of the human immunophilin FKBP-12 complexes with FK506 and rapamycin.. J Mol Biol.

[21] Heinemann U, Büssow K, Mueller U, Umbach P (2003). Facilities and methods for the high-troughput structure analysis of human proteins.. Acc Chem Res.

[22] Otwinowski Z, Minor W (1997). Processing of X-ray diffraction data collected in oscillation mode.. Methods Enzymol.

[23] Adams PD, Grosse-Kunstleve RW, Hung LW, Ioerger TR, McCoy AJ (2002). PHENIX: building new software for automated crystallographic structure determination.. Acta Crystallogr D.

[24] Cohen SX, Ben Jelloul M, Long F, Vagin A, Knipscheer P (2008). ARP/wARP and molecular replacement: the next generation.. Acta Crystallogr D.

[25] Emsley P, Cowtan K (2004). Coot: model-building tools for molecular graphics.. Acta Crystallogr D.

[26] Murshudov GN, Vagin AA, Dodson EJ (1997). Refinement of macromolecular structures by the maximum-likelihood method.. Acta Crystallogr D.

[27] Lovell SC, Davis IW, Arendall WB, de Bakker PI, Word JM, Prisant MG, Richardson JS, Richardson DC (2003). Structure validation by Cα geometry: φ, ψ and Cβ deviation.. Proteins.

[28] DeLano WL (2003). The PyMOL Molecular Graphics System.

[29] Baker NA, Sept D, Joseph S, Holst MJ, McCammon JA (2001). Electrostatics of nanosystems: application to microtubules and the ribosome.. Proc Natl Acad Sci US.

[30] Larkin MA, Blackshields G, Brown NP, Chenna R, McGettigan PA (2007). Clustal W and Clustal X version 2.0.. Bioinformatics.

